# Vitamin D Status of the British African-Caribbean Residents: Analysis of the UK Biobank Cohort

**DOI:** 10.3390/nu13114104

**Published:** 2021-11-16

**Authors:** Rebecca M. Vearing, Kathryn H. Hart, Karen Charlton, Yasmine Probst, David J. Blackbourn, Kourosh R. Ahmadi, Susan A. Lanham-New, Andrea L. Darling

**Affiliations:** 1 Faculty of Health and Medical Sciences, School of Biosciences and Medicine, University of Surrey, Guildford GU2 7XH, UK; k.hart@surrey.ac.uk (K.H.H.); d.blackbourn@surrey.ac.uk (D.J.B.); k.ahmadi@surrey.ac.uk (K.R.A.); s.lanham-new@surrey.ac.uk (S.A.L.-N.); a.l.darling@surrey.ac.uk (A.L.D.); 2 Faculty of Science Medicine and Health, School of Medicine, University of Wollongong, Wollongong, NSW 2522, Australia; karenc@uow.edu.au (K.C.); yasmine@uow.edu.au (Y.P.); 3 Illawarra Health and Medical Research Institute, Wollongong, NSW 2522, Australia; 4 Institute of Medical Sciences, University of Aberdeen, Foresterhill, Aberdeen AB25 2ZD, UK

**Keywords:** vitamin D, 25(OH)D, African-Caribbean, Afro-Caribbean, UK Biobank, diet, supplement, skin type

## Abstract

The vitamin D status of the United Kingdom (UK) African-Caribbean (AC) population remains under-researched, despite an increased risk of vitamin D deficiency due to darker skin phenotypes and living at a high latitude. This cross-sectional study explored the vitamin D status and intake of AC individuals (*n* = 4046 with a valid serum 25(OH)D measurement) from the UK Biobank Cohort, aged ≥40 years at baseline (2006–2010). Over one third of the population were deficient (<25 nmol/L), 41.1% were insufficient (25–50 nmol/L) and 15.9% were sufficient (>50 nmol/L). Median (IQR) 25(OH)D was 30.0 (20.9) nmol/L. Logistic regression showed that brown/black skin phenotype, winter blood draw, not consuming oily fish and not using vitamin D supplements predicted increased odds of vitamin D deficiency, whilst older age and a summer or autumn blood draw were significantly associated with reduced odds of vitamin D deficiency. Vitamin D deficiency and insufficiency were prevalent in this AC population and is of considerable concern given the individual and societal implications of increased morbidity. Public health messaging for this group should focus on year-round vitamin D supplementation and increasing intakes of culturally appropriate vitamin D-rich foods. These data also support the urgent requirement for a revised vitamin D RNI for ethnic groups.

## 1. Introduction

Vitamin D is formed when ultraviolet-B (UVB) rays are in contact with human skin [1]. Vitamin D can also be sourced through foods such as oily fish, UV-exposed mushrooms and fortified products, as well as nutritional supplements [2]. However, the majority is obtained through conversion of dehydrocholesterol to vitamin D_3_ in the skin following sunlight exposure [3,4,5,6]. Vitamin D is essential for calcium homeostasis; and, therefore, the maintenance of musculoskeletal health [7,8]. Beyond this association, links have been found between vitamin D deficiency and certain cancers, multiple sclerosis, diabetes, and other health conditions; although the strength of these associations remains unclear [7,9,10,11,12]. Maintaining good vitamin D status should be encouraged for both musculoskeletal health and non-musculoskeletal health benefits.

The gold standard method for assessing vitamin D status is serum 25(OH)D level [13]. Vitamin D insufficiency (<50 nmol/L) remains a global health issue, negatively impacting individual health and placing a burden on health care systems [14,15]. At higher latitudes, dietary intake should be monitored, since the skin is unable to synthesise vitamin D in the winter and, once stores are depleted, vitamin D status is determined solely by dietary intake [16,17,18]. 

Individuals living in the United Kingdom (UK), particularly ethnic minorities, are at high risk of vitamin D deficiency [17]. This is likely due to high latitude and to skin type, which are amongst the array of factors that contribute to vitamin D status [5,16,18,19,20]. The African-Caribbean (AC) UK population, specifically, are likely to be at high risk of vitamin D deficiency. This group refers to those with African ancestry who migrated to other countries via the Caribbean, or those native to the Caribbean with African ancestry [21]. Due to darkly pigmented skin, and thus increased melanin content, the capacity to synthesise vitamin D from sunlight is reduced in this population [18,22]. This is particularly concerning at high latitudes, due to the relatively low sunlight hours year-round, and an inability to synthesise vitamin D during winter months [16,17,18] due to the zenith angle of the sun. This population group is considerably under researched in the area of vitamin D, both at high latitudes and at low latitudes, the latter being the group’s ancestral origin. 

High latitude AC research is inadequate, and the current extent of vitamin D deficiency is unknown. Reasons include relatively small sample sizes, improvements in quantification and in public policy guidance. Three studies found a high prevalence of vitamin D deficiency [23,24,25], including one UK study by Ford et al. (2006) that found one in four AC participants were deficient at the end of summer [25]. This is concerning, as 25(OH)D levels are expected to be at their peak after the summer months and suggests that summer sunlight hours in the UK may not be sufficient for those with darker skin to obtain the required levels of vitamin D for musculoskeletal and general health [25]. The UK Scientific Advisory Committee on Nutrition (SACN) suggests suggested that this population should consider vitamin D supplementation year-round [16]. As in all UK population groups, poor vitamin D intake is common in AC. For example, Donin et al. (2010) [26] found their AC populations consumed 1.72 ± 1.2 µg/day on average, well below the UK recommended nutrient intake (RNI) of 10 µg/day [16]. This is likely to be due to small amounts of the vitamin being naturally present in foods regularly consumed in the UK. Conversely, Castaneda-Gameros et al.’s (2018) [27] AC population had a mean intake of 9.63 ± 1.2 µg/day but was skewed by the use of vitamin D supplements by some participants. 

In the present study, we aimed to update the evidence in terms of the extent of vitamin D deficiency in the AC population, who are at high risk of deficiency, and address the literature gap in this under-researched population by analysing data on serum 25(OH)D concentrations and vitamin D dietary intakes in the largest sample of AC individuals to date. We used data from the AC subset of the UK Biobank, which is a UK-wide cohort investigating health in middle and older life [28]. To our knowledge, this is the largest epidemiological analysis to date on the adult British AC population in relation to vitamin D. We also completed a sub-analysis of the UK Biobank African (AF) population in order to assess whether the findings and recommendations from the AC population are likely to translate to other Black populations at high latitudes (UK and other countries). The findings for AC and AF were also compared to South Asian (SA) and White European (WE) UK Biobank participants to provide specific context. 

## 2. Materials and Methods

### 2.1. UK Biobank

The UK Biobank Cohort (www.ukbiobank.ac.uk/ accessed on 1 March 2021) contains data on the health and well-being of a large UK-wide cohort of over 502,000 individuals [28]. The UK Biobank aims to investigate factors which impact health outcomes in middle and older life by following the health of participants over time [29]. Participants were initially recruited through the National Health Service (NHS) between 2006 and 2010 [28]. They attended assessment centres at various latitudes (51.4° N to 56.0° N) across the UK and were aged 40 years or over at their baseline visit. 

All participants who self-identified as AC (*n* = 4517), AF (*n* = 3394), SA (*n* = 8023) and a subsample of the WE population (*n* = 4446) were eligible for inclusion in this study. The sample of WE individuals was randomly selected using SPSS (v 27 (Chicago, IL, USA) to ensure that sample sizes for the ethnic groups were comparable, considering that the WE subset of the UK Biobank includes over 450,000 individuals. 

This cross-sectional analysis only included participants with a valid serum 25(OH)D measurement, specifically *n* = 4046 AC, *n* = 2993 AF, *n* = 7256 SA and *n* = 3986 WE (total *n* = 18,281). Over 33.0% of AC, 28.0% of AF, 28.0% of SA and 39.6% of WE also had data for dietary vitamin D intake. With the exception of vitamin D and calcium intakes (see diet section below), all analyses utilised baseline values only. Bonferroni correction of *p*-values were undertaken when between ethnicity post-hoc tests were conducted.

### 2.2. Serum 25(OH)D Concentration

The primary outcome of this study was serum 25(OH)D concentration, reported as median (IQR). We also assessed the percentage of individuals within different 25(OH)D evidence-based cut-off points: <25 nmol/L deficient (15); >25–50 nmol/L insufficient [30] and >50 nmol/L sufficient [30]. 

Serum 25(OH)D was measured once at baseline through a non-fasted blood draw during any season of the year. Serum 25(OH)D concentration was determined using DiaSorin Liason XL, a direct competitive chemiluminescent immunoassay [31]. This assay measures vitamin D synthesised from sunlight exposure, as well as animal (vitamin D_3_) and non-animal (vitamin D_2_) dietary sources. Both D_3_ and D_2_ were included in the reported 25(OH)D measurement. The lower limit of detection for the assay was 10 nmol/L. It is important to note that this assay underestimates levels of 25(OH)D by 4% at 25 nmol/L and overestimates by 5–10% when 25(OH)D levels are >40 nmol/L [32].

As a result of having an undetectable 25(OH)D measurement (<10 nmol/L), some participants (*n* = 937) did not have a 25(OH)D measurement. To reduce potential bias in our analysis, missing values were assigned a correction of lower limit/square root(^2^) as their 25(OH)D measurement (i.e., 7.1 nmol/L) [33]. No 25(OH)D measurements were considered outliers (>300 nmol/L), therefore, all measurements were valid for analysis. Further details on serum 25(OH)D measurement can be viewed on the UK Biobank website https://biobank.ctsu.ox.ac.uk/crystal/field.cgi?id=30890 accessed on 1 March 2021 [29].

### 2.3. Dietary Data

Participants who had their baseline visit from February 2011 to April 2012 completed a 24 h dietary recall questionnaire (Oxford WebQ) via touchscreen. Participants who attended before February 2011 were not offered this at baseline. However, all participants who had provided investigators with their email addresses were invited to complete the questionnaire online a further four times [34]. Therefore, participants may have completed the questionnaire up to five times, with each questionnaire being requested on different days of the week to capture intake on weekdays compared to weekends.

The Oxford WebQ questionnaire has been previously validated against other 24 h recalls, comparing within a 10% difference or less between the methods [35]. Participants are asked to report their frequency of consumption of 200 different food and drinks in the preceding 24 h, for example, ‘did you eat any bread or crackers yesterday?’ [34]. These were yes/no questions, and positive answers would result in an expansion of the question, for example, the participant would then answer the amount of bread or crackers consumed in the previous day. Questions on typical serving size, typical dietary intake and special diets were also asked. The following food groups were included: hot and cold beverages; alcoholic beverages; cereal; milk, eggs and cheese; bread, pasta and rice; soups, snacks and pastries; meat and fish; vegetarian alternatives; spreads, sauces and cooking oils; fruit and vegetables; meal types and vitamin and mineral supplements [34]. Nutrient intakes, including vitamin D and calcium, were calculated by the UK Biobank by multiplying the quantity consumed by the participant by the nutrient composition of the food or beverage, according to McCance and Widdowson’s Composition of Foods [35,36], median intakes for both nutrients were calculated by the University of Surrey research team dependent on how many times they completed the 24 h dietary recall. See http://biobank.ctsu.ox.ac.uk/crystal/crystal/docs/DietWebQ.pdf (accessed on 3 March 2021) for further details. To assess vitamin D and calcium intake, we calculated the overall median intake across all recalls reported at baseline and online follow ups. 

A general touchscreen food frequency questionnaire (FFQ) was completed at baseline by all participants to assess longer term food consumption of foods per day or over a week depending on the food. The questionnaire recorded frequency of food groups consumed, including meats, fish and oily fish, fruits and vegetables, dairy, grains and spreads. It also asked questions about major changes in the diet and week to week variations. This data was used to assess oily fish intake and to create a dummy variable to code whether participants were vegetarian or not, based on the participant answering ‘never consumes’ to the following foods: oily fish, non-oily fish, processed meat, poultry, pork, beef, lamb and mutton. This new variable was chosen over a special diets question about vegetarianism, as almost all Biobank participants had data for the food categories, resulting in a more accurate representation and few participants answered the specific question for vegetarianism, and deemed less reliable. See http://biobank.ctsu.ox.ac.uk/crystal/crystal/docs/TouchscreenQuestionsMainFinal.pdf (accessed on 4 April 2021) for further details of the FFQ items.

Individuals reporting use of any vitamin D containing supplement, (including single vitamin D, combined calcium and vitamin D, multivitamins, and combined multivitamin and mineral) were dummy coded as a user of a vitamin D containing supplement. We did not include mineral-only supplements. We could not include vitamin D from cod-liver oil supplements as the fish oil question in the UK Biobank did not specify whether the person was taking omega fish oil or cod liver oil. Full details of the dummy variable created can be found in the supplementary file of Darling et al., 2018 [14].

### 2.4. Statistical Analysis

Statistical analysis was also conducted using SPSS software, while figures and graphs were created using GraphPad Prism (v8, San Diego, CA, USA). Chi-squared (χ^2^) tests were used to test for associations of categorical outcomes with sex and ethnicity. Vitamin D intake and 25(OH)D concentration were not normally distributed and, therefore, were analysed using non-parametric tests. This was important for vitamin D intake because using a log-transformation would cause loss of data for those participants who consumed 0 µg of vitamin D, a valid intake. The one exception to the use of non-parametric tests was the use of a two-way ANOVA to assess 25(OH)D by region, season and ethnic-gender group. The decision was made to use this test, rather than a larger number of non-parametric alternatives, to avoid multiple testing and the increased risk of a type 1 error.

A binominal logistic regression analysis was performed on the AC population to explore the predictors of vitamin D deficiency (<25 nmol/L). A dummy variable was created for 25(OH)D (coded as those with 25(OH)D measurements ≥25 nmol/L vs. <25 nmol/L). In all models the first category was set as the reference. Continuous variables such as age and BMI were recoded into categorical variables to aid interpretation. For model 1, the variables sex, ethnicity, age, and BMI were added to the model). Next, oily fish intake and vitamin D supplementation were added, followed by region, season of blood draw and gross annual household income ). Vegetarianism and vitamin D intake were not included in the final models due to low number of counts in one category or a large amount of missing data, respectively. Results were presented as OR (95% CI) unless otherwise stated.

## 3. Results

### 3.1. Descriptive Characteristics 

**Figure 1:**
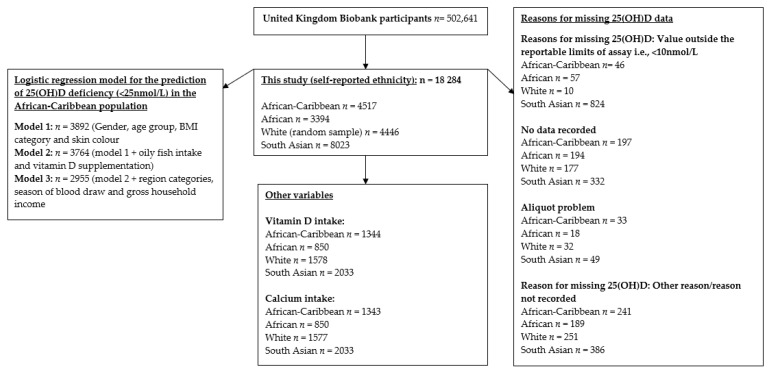
Flow chart of the UK Biobank participants included in the analyses.

Detailed participant characteristics for the *n* = 4046 AC and *n* = 2993 AF participants with valid 25(OH)D data are shown in Table 1, by ethnicity and gender. See Figure 1 for the flow of participants from the UK Biobank sample compared to those included in the analyses.

### 3.2. Descriptive Data: Categorical Variables for the African-Caribbean and African Participants

The majority of both the AC and AF participants were in the 40–60-year-old age group, with a slightly higher proportion of AC than AF participants in the >60-year-old age group. There was an association between ethnicity and BMI, with the AF population having a higher percentage of obesity than the AC group (42.4% vs. 38.7% obese, respectively, *p* < 0.001). There was also an association between gender and BMI, with obesity being more prevalent in females for both AC (43.0% vs. 31.5% *p* < 0.001) and AF (56.3 vs. 29.0% *p* < 0.001). For self-reported health, there was an association with both gender (*p* = 0.03 AC and *p* < 0.001 AF) and ethnicity (*p* < 0.001), with more AFs (70.1%) reporting excellent/good health compared to ACs (58.0%). For both ethnicities, most blood draws were carried out in spring, followed by summer, autumn, and winter. 

Less than 1% of both AC and AF participants self-reported they followed a vegetarian diet pattern. Taking a vitamin D containing supplement was associated with both ethnicity (*p* < 0.001) and gender (*p* < 0.001 AC and AF). Females in both ethnic groups more commonly took a vitamin D containing supplement compared to males, while in total, slightly more AC (37.9%) than AF (31.6%) participants did so. There was an association between ethnicity and oily fish intake (*p* < 0.001), with the most common intake being once a week for AC and AF (37.2% and 32.0% respectively). An association was also found between oily fish intake and gender within AF (*p* = 0.01) and AC (*p* < 0.001), although as the gender differences were very small, they were likely not biologically relevant.

There was an association between ethnicity and skin colour (*p* < 0.001), with 93.0% of AC and 88.2% of AF’s reporting having either brown or black skin phenotype. For AC, 60.2% reported their ease of skin tanning to be ‘get very or moderately tanned’, whilst only 6.6% reported ‘never tan, only burn’. Within AF participants, 24.2% reported that there was an association between time spent outdoors in summer and ethnicity (*p* < 0.001). The category with the highest percentage of people was ≥5 h (39.0% AC and 36.5% AF), whilst <1 h time spent outdoors was reported in 5.9% of AC and 8.2% of AF participants. For sun protection, there was also an association with ethnicity (*p* < 0.001). The category with the highest percentage of people from both ethnic groups was ‘never or rarely used sun protection’ (40.9% AC and 66.2% AF). A lower percentage of individuals reported using sun protection ‘always or most of the time’ (20.4% AC and 6.3% AF). A very low percentage in both groups reported not going out in the sunshine (1.6% AC and 2.4% AF).

There was a significant association between ethnicity and income (*p* < 0.01). In both ethnic groups, across both genders, the highest percentage of participants fell within the <£18,000 per year income category. Fourteen-point two percent of AC’s and 12.4% of AF’s reported an income of ≥£52,000 per year. There was a statistically significant association between geographical region and ethnicity (*p* < 0.001), but actual percentage differences in each region between AC and AF were negligible. Over half of all participants from AC and AF were living in London or Southern England. There was also an association between place of birth and ethnicity (*p* < 0.001), with 58.1% of the AC participants and 88.8% of the AF participants reporting they were not born within the UK or ROI. 

A small percentage of females in each group were using oral contraceptives, but it was associated with ethnicity (3.2% AC and 2.3% AF, *p* < 0.001), albeit the percentage difference was small. Almost half of ACs and AFs reported being post menopause (40.9% and 46.6%, respectively). Tobacco smoking was associated with ethnicity (*p* < 0.001) and with gender in each ethnic group (*p* < 0.001 AC and AF). A minority of participants within each ethnic group reported using tobacco, with males reporting higher use than females (23.8% vs. 12.5% in AC and 10.4 vs. 3.2% in AF). 

### 3.3. Reasons for Missing 25(OH)D Data

For 25(OH)D, *n* = 937 participants’ measurements were under the assay detection limit and, therefore, were recoded to 7.1 nmol/L. These participants comprised of *n* = 46 AC (1.0%), *n* = 57 AF (1.7%), *n* = 824 SA (10.3%) participants and *n* = 10 WE (0.2%). In the WE and AC groups, a higher percentage of females had a 25(OH)D measurement under the detection limit compared to males (70% vs. 30% WE, 69.6% vs. 30.4% AC), whilst in the SA and AF groups males reported a higher number of undetectable readings than females (50.5% vs. 49.5% SA, 61.4% vs. 38.6% AF). Other reasons for missing 25(OH)D data included no data recorded, aliquot problem, UK Biobank researcher deeming the result not reportable for reason other than above or below the detectable limit and ‘missing’. Data are shown in Figure 1.

### 3.4. Primary Outcomes

#### 25(OH)D Concentration

**Figure 2:**
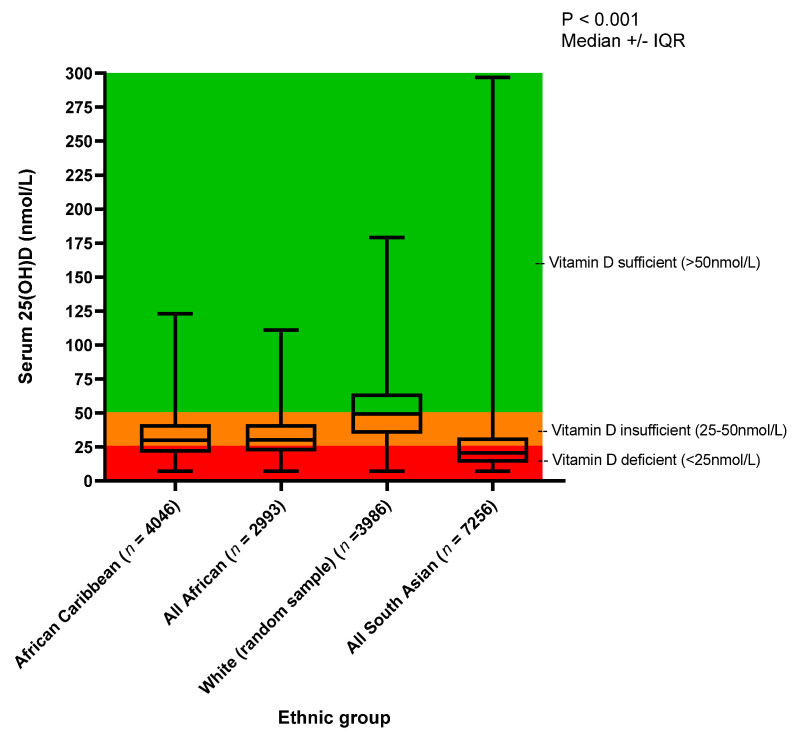
The median (IQR) serum 25(OH)D concentration for the African Caribbean (*n* = 4046), African (*n* = 2993), White (random sample) (*n* = 3986) and the South Asian (*n* = 7256) participants from the UK Biobank. *p*-Value was analysed using Kruskal–Wallis H test for African-Caribbean vs. African vs. White vs. South Asian.

Results for the analysis of 25(OH)D concentration are shown in Table 2 and Figure 2. In all ethnic groups, except the SA group, a higher percentage of participants recorded an insufficient (25–50 nmol/L) than either a deficient (<25 nmol/L) or sufficient (>50 nmol/L) 25(OH)D measurement. In all groups, except the SA group, the median 25(OH)D measurement fell within the vitamin D insufficient category. The WE population had the highest median 25(OH)D concentration (49.2 ± 29.5 nmol/L), followed by the AF (30.2 ± 20.0 nmol/L) and AC groups (30.0 ± 20.9 nmol/L), and lastly the SA population (20.7 ± 18.5 nmol/L) (*p* < 0.001 between ethnicities). Using a Bonferroni adjusted *p*-value cut-off (*p* < 0.008), there was a significant difference in 25(OH)D between each pair of ethnic groups (*p* < 0.001), except between the AC and AF groups (*p* = 0.41). 

Within the AC group, a Mann–Whitney test showed a gender difference in 25(OH)D (*p* = 0.01), with a median (IQR) of 29.5 nmol/L (19.3 nmol/L) for males, and 30.4 nmol/L (21.5 nmol/L) for females, albeit the 25(OH)D difference was small. A higher percentage of males (40.3%) were vitamin D deficient (<25 nmol/L) compared to females (35. 1%), and conversely, a higher percentage of females were sufficient (16.7%) (>50 nmol/L) compared to males (14.5%). Similarly, for AF, a Mann–Whitney test showed a gender difference for 25(OH)D (*p* < 0.001), with more males (38.3%) having a deficient vitamin D measurement (<25 nmol/L) compared to females (31.1%). Very few AF males or females (11.3% and 16.3% respectively) recorded sufficient vitamin D levels (>50 nmol/L).

**Figure 3:**
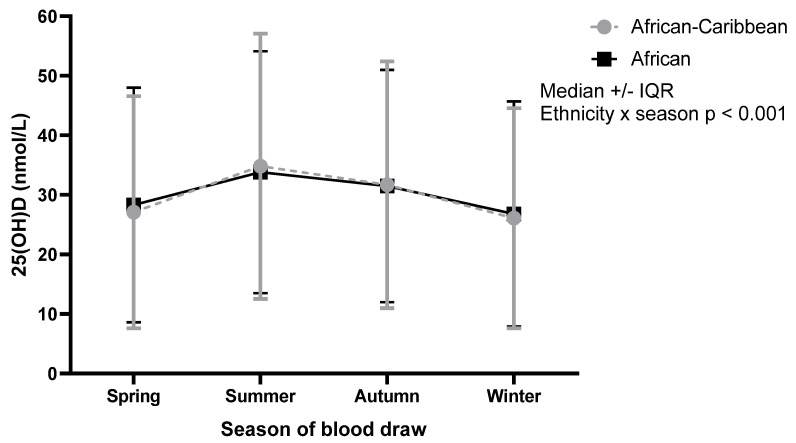
Concentration of 25(OH)D by season. Note: Each participant has one measurement in one season, therefore this graph does not show repeated measures. There was a statistically significant interaction between ethnicity and season. Both groups reported the highest median 25(OH)D concentrations in summer and the lowest in winter. *p*-Values from two-way ANOVA.

Comparing the ethnic groups, the WE and AC groups (15.9% each) recorded the highest percentage of participants with sufficient vitamin D levels (>50 nmol/L). The SA group on average was vitamin D deficient (<25 nmol/L), with a median (IQR) of 20.7 (18.5) nmol/L, with 25(OH)D statistically significantly, but not clinically, different in males (20.1 (18.5) nmol/L) and females (21.8 (21.1) nmol/L) (*p* < 0.001). In the SA group as a whole, 61.4% were vitamin D deficient (<25 nmol/L), with only 7.2% sufficient (>50 nmol/L). There was a significant association between season of blood draw and median 25(OH)D concentration (*p* < 0.001). In both AC and AF, the highest median 25(OH)D concentration was in summer, followed by autumn, spring and winter (Table 3 and Figure 3). The median 25(OH)D concentration for those who were measured in spring, summer and autumn were considered vitamin D insufficient (>25–50 nmol/L). The winter measurements were insufficient but on average were near the deficiency line (<25 nmol/L).

A two-way ANOVA showed a significant association between ethnicity, geographical location and median 25(OH)D (*p* < 0.001) (Table 4). Across all ethnicities and geographical locations, 25(OH)D measurements, on average, were insufficient. AC and AF participants residing in London/South England, had the highest median 25(OH)D levels (31.0 nmol/L and 32.3 nmol/L respectively), followed by those in Scotland/North England (30.1 nmol/L and 27.4 nmol/L respectively). Those living in Midlands/Wales were borderline deficient (27.5 nmol/L for AC and 25.9 nmol/L for AF). However, differences in 25(OH)D between ethnicities and regions were small.

### 3.5. Prediction of 25(OH)D Deficiency: Logistic Regression Model

A binominal logistic regression was performed in AC to ascertain the ability of age, gender, BMI, skin colour, oily fish intake, vitamin D supplement use, region, season of blood draw and income to predict the odds of vitamin D deficiency (<25 nmol/L) (See Table 5). 

Each logistic regression model was significant (*p* < 0.001). In the final model, younger age, darker skin colour, not consuming oily fish, non-use of vitamin D containing supplements and living in Midlands/Wales were significant predictors of higher odds of being vitamin D deficient. Blood draws in summer and autumn were significantly associated with a reduction in odds of having vitamin D deficiency (*p* < 0.001).

Specifically, younger age (40–69 years) was associated with increased odds of vitamin D deficiency compared with older age (>69 years) (OR of 0.44 (0.37–0.53). There was a significant association between having a darker skin type and vitamin D deficiency, with Brown/Black having 1.77 times higher odds of having deficiency compared to those with other skin types. Those who never consumed oily fish were significantly more likely to be vitamin D deficient (OR 1.74 (1.19 to 2.54) compared to those did consume oily fish (reference value). A significant association was found between vitamin D containing supplement use and vitamin D deficiency, with non-users of vitamin D supplementation being almost three times more likely to be vitamin D deficient (OR of 2.97 (2.50 to 3.53) compared with users. A winter blood draw showed 1.21 times higher odds (OR of 1.21 (0.98 to 1.51) of vitamin D deficiency when compared to blood draws in spring (reference value). Blood draws in autumn (OR of 0.63 (0.51 to 0.74) and summer (OR of 0.36 (0.28 to 0.45) were also associated with a reduction in odds of having vitamin D deficiency. 

Compared with those from London/South England (reference value), those residing in Midlands/Wales had 1.22 times higher odds (OR of 1.22 (1.01 to 1.46; *p* = 0.04), but results were not significant for Scotland/North England (OR of 1.15 (0.92 to 1.43; *p* = 0.22). Gender and income were not associated with odds of deficiency. Overweight (25–29.5 kg/m^2^) or obese BMI (>30 kg/m^2^) did not predict vitamin D deficiency, compared with normal/underweight.

### 3.6. Dietary Intake

All ethnic groups had a suboptimal intake of vitamin D (<10 µg/day). There was a significant difference between the ethnic groups as a whole for median vitamin D intake (Kruskal–Wallis *p* < 0.001). The median vitamin D intake for the AC group was 1.6 µg/day, with only 4.8% of the population meeting the UK adult recommended nutrient intake of 10 µg/day [16]. Likewise, the AF population had a median intake of 2.1 µg/day, with 90.9% of the group having a suboptimal dietary vitamin D intake (<10 g/day). The WE group had a median intake of only 1.9 µg/day, meaning 95.9% had suboptimal intake. The lowest intake was in the SA group, with a median intake of 1.1 g/day, and 97.0% of participants with suboptimal intake (Table 6). 

A Kruskal–Wallis test showed a significant difference between the ethnic groups for median calcium intake (*p* < 0.001). The median calcium intake in all groups exceeded the recommendation of 700 mg/day of calcium [16]. The WE group had the highest number of participants with an optimal intake (>700 mg/day) at 78.6%, followed by the SA group (63.3%), the AF (52.5%) and AC (52.3%) groups.

## 4. Discussion

This study aimed to update the evidence on levels of vitamin D deficiency in this under researched ethnic population. The UK Biobank AC participants were found to be vitamin D insufficient on average, with median vitamin D intakes only meeting 4.8% of the UK recommendation. The AF group was also 25(OH)D insufficient, with almost identical median values to AC. This suggests that our results for the UK AC population may be comparable to other Black populations residing in the UK, and, therefore, recommendations may be similar for each group. It is important, however, to continue research on specific Black populations, to acknowledge the differences in genetics, cultural, behavioural and environmental factors that impact on these heterogenous groups [21].

Our findings support the results of a recent meta-analysis, which found vitamin D insufficiency across four small (total *n* = 995) observational studies [23,24,25,37] of AC populations living at high latitudes [38]. In the current analysis, the AC group had a lower 25(OH)D concentration than the WE group, who were borderline 25(OH)D sufficient on average. This was as expected, with research showing those with lighter skin types living at higher latitudes have a higher 25(OH)D concentration, in comparison to their counterparts with darker skin types [5,20,39]. It is important to note, that there may be biological differences between racial groups in relation to vitamin D metabolism [30]. It has been found that some population groups have genetic adaptations to being exposed to lower levels of sunlight [40,41]. For example, Inuit populations have been found to convert vitamin D to its active form, calcitriol, more efficiently despite living at a high latitude [40]. Whether these genetic adaptions translate to African Caribbean populations is currently unknown. 

The AC group had a higher 25(OH)D concentration than the SA group. We speculate that due to a larger number of Hindu, Sikh and Muslim individuals in the UK SA population compared with UK AC population, that more UK Biobank individuals in the SA group have a covered dress style than do the UK Biobank AC, although the UK Biobank did not ask about this specifically. Some research has suggested that SA women also tend to avoid the sun [42] and have a low usage of vitamin D supplementation [43]. This has not been reported for AC to our knowledge. These factors, along with other personal and environmental factors, may explain the 25(OH)D difference seen here between AC and SA.

Finally, our findings agree with the Health Survey for England (2010), which did not analyse the AC population separately, but found their White population had the highest median 25(OH)D, followed by the Black and then Asian adults [44]. These data also support the urgent requirement for a revised vitamin D RNI for ethnic groups due to higher levels of deficiency and justifies undertaking a clinical trial to evaluate the impact of supplementing vitamin D in the AC population.

In our current study, for AC, vitamin D insufficiency was present in every season of the year, with borderline deficiency in winter and spring. Vitamin D deficiency and insufficiency during the winter months in the UK can largely be attributed to the lack of cutaneous synthesis of vitamin D due to the zenith angle of the sun [16,17]. However, insufficiency in summer, when 25(OH)D levels should be at their peak, is concerning. This is surprising considering 39% of the AC population reported spending ≥5 h per day in the sun in summer and 40.9% never or rarely used sunscreen. These findings suggest that even during the summer, UVB levels may not be adequate to increase serum 25(OH)D levels to sufficient levels in ethnic minorities, perhaps as a result of their skin tone [16,18,22] which acts as a barrier to UVB irradiation.

It is important to note that vitamin D status is multifactorial, and skin type is not the only contributor to vitamin D status. The AC diaspora who migrated away from the equator to a much higher latitude have become at high risk for vitamin D deficiency. It is well known that there is an inverse association between distance from the equator and 25(OH)D concentration [38]. As shown in our results for 25(OH)D in AC and AF, median 25(OH)D concentration was insufficient at all UK sites. There was a statistical interaction between ethnicity and geographical location, but actual regional differences within ethnicity were small (up to 3 nmol/L) for most comparisons. The one exception was the Midlands/Wales, whose residents were up to 6 nmol/L lower than those in London/Southern England. 

Vitamin D intake was well below the RNI of 10 µg/day for not only the AC group, but all ethnicities analysed in this paper, which is consistent with other research in the UK showing suboptimal intakes in AC [26,27,38] and SA [14,43,45] populations and confirming that diet alone is currently an inadequate source of vitamin D in the UK.

Multiple health outcomes have been associated with vitamin D deficiency. In the short term, vitamin D insufficiency can cause fatigue, bone and muscle pain [16,18]. Longer term insufficiency is known to lead to osteopenia or osteoporosis and accompanying increased risk of fractures [16,18]. Vitamin D insufficiency may also be associated with non-musculoskeletal health conditions such as seasonal affective disorder, neurocognitive dysfunction, type 2 diabetes, and cardiovascular disease [18,22,46]. This suggests AC, AF and SA populations living in the UK and other higher latitude countries are at significant risk of excess morbidity, with a need to increase 25(OH)D concentrations in these groups.

Vitamin D supplementation, in combination with intake of vitamin D fortified foods and foods naturally rich in vitamin D, should be considered year-round for these ethnic groups. This is extremely important, as sourcing vitamin D from food and supplements becomes essential when sun exposure is insufficient for cutaneous synthesis of vitamin D. Toxicity from vitamin D supplementation can occur, although rare [47]. A dosage of 10 µg/day, recommended by the Scientific Advisory Committee on Nutrition (SACN), is a safe level to consume year-round, as this concentration takes into consideration both dietary vitamin D intake and UVB exposure [16]. Ten micrograms per day is calculated to be sufficient for 97.5% of the UK population to attain a serum 25(OH)D concentration >25 nmol/L year-round, which reduces the risk of poor musculoskeletal health [16]. Due to lack of research, it is unknown whether Asian and Black groups have a higher requirement for vitamin D to achieve the same blood levels and/or health benefits.

Our logistic regression results suggest that younger age and darker skin colour predict increased odds of vitamin D deficiency in the AC population. This was unexpected as there is an age-related reduction in 7-dehydrocholesterol, which converts to vitamin D3 when in contact with UVB [18,48], and it is predicted that older people should have lower 25(OH)D than middle-aged people. However, our result suggests that there are a range of factors at play. For example, the younger age group in the UK Biobank may be indoor workers and may therefore be exposed to less sunlight compared to an older, retired population that has more time for outdoor leisure activities. The younger age group were only 10–20 years younger so the age differential in 25(OH)D production may be small. As expected, due to the ability of melanin to filter our UVB radiation, darker skin colour was associated with a higher odds of vitamin D deficiency. Gender was not a predictor of vitamin D deficiency, with only a 1 nmol/L difference in 25(OHD) difference between genders. 

Not consuming oily fish and not taking vitamin D supplements were associated with increased odds of vitamin D deficiency. This reinforces our recommendation that further public health messages are needed, especially for ethnic minority groups, to promote vitamin D supplementation and intake of foods naturally rich in vitamin D (e.g., oily fish, eggs), or fortified with vitamin D (e.g., breakfast cereals), as is culturally appropriate for each individual.

Obesity was not found to be associated with odds of deficiency in AC. This is surprising given the known negative association between 25(OH)D and adiposity [49]. Indeed, 25(OH)D may be sequestered in adipocytes [16,50,51] although the effect of weight loss on release of 25(OH)D into the systemic circulation is still unknown [30,51]. Maintaining a healthy weight is an effective public health message to not only assist in the reduction of vitamin D deficiency, but also weight associated conditions such as type 2 diabetes and cardiovascular disease [16] which are particularly common in AC, AF and SA populations. 

Blood draws in autumn and summer were associated with a reduction in odds of having vitamin D deficiency, which was expected as cutaneous synthesis of vitamin D is only possible during the summer months. General public health messages to increase sunlight exposure in the summer months may be too simplistic, as there are a myriad of complex factors that impact on cutaneous synthesis, as well as the need to balance sunlight exposure for cutaneous synthesis with the adverse health outcomes associated with over exposure [16] and consideration of cultural factors which may limit a person’s ability to expose skin to the sun. After adjustment for confounders in the regression model, living in Midlands/Wales was associated with an increased odds of vitamin D deficiency (<25 nmol/L) in comparison to London/South England, but living in Scotland/Northern England did not. Across all geographical regions vitamin D deficiency was still an issue in AC, as well as the other ethnic groups, and so precautions should be taken across all UK areas. 

Very little research has assessed the vitamin D status of the AC population living at higher latitudes. To the authors’ knowledge, this is the largest epidemiological analysis to date on the adult UK AC population in relation to vitamin D. Previous research, including in the UK Biobank, did not assess AC and AF population separately. It is important to do so, due to the differences between Black populations which may impact on their vitamin D status. 

The use of the DiaSorin Liaison XL assay to measure 25(OH)D is a potential limitation as it can underestimate 25(OH)D [32], in comparison to the gold standard for 25(OH)D measurement which is Liquid-chromatography-tandem-mass-spectrometry (LC-MS/MS) [52] The Biobank aimed to minimise this bias (See https://biobank.ndph.ox.ac.uk/showcase/ukb/docs/biomarker_issues.pdf accessed on 10 November 2021). Further, the vitamin D deficiency cut-offs used are not population specific, and whether they are suitable for the African Caribbean population, currently remains unknown. Further research is needed to explore whether this population living at high latitudes show clinical symptoms such as osteoporosis or osteomalacia at low levels of vitamin D deficiency. A parametric two-way ANOVA test was used on non-parametric data for the analysis of geographical region, season, and ethnic group/gender for median 25(OH)D, but we considered this to be more favourable than doing multiple Mann—Whitney analysis, which carries the risk of multiple testing and increased risk of a type 1 error. The assessment of diet was limited by the use of a FFQ and 24 h dietary recall, which are not considered as accurate as diary assessment methods such as four-day food diaries with weighed portion sizes or photograph assisted portion estimation. Finally, the UK Biobank participants may be more health conscious than the national average, meaning that these results may be an underestimate of the true degree of vitamin D deficiency in the UK AC population.

Future research in this population must include clinical trials on the level of vitamin D deficiency in this population group as well clinical symptoms of osteomalacia and osteoporosis through measuring bone density. Findings would help form recommendations for supplementation in this group [16]. 

## 5. Conclusions

The median 25(OH)D concentration of the UK Biobank AC population was 30.0 nmol/L (IQR 20.9 nmol/L), with 37.0% considered vitamin D deficient (<25 nmol/L), 41.1% insufficient (>25–50 nmol/L) and only 15.9% sufficient (>50 nmol/L). Vitamin D intake in AC was found to be suboptimal, with a median intake of only 1.6 µg/day (IQR 2.6 µg/day) and only 4.8% of the population meeting the UK RNI of 10 µg/day. Younger age, darker skin type, not using vitamin D supplementation, not consuming oily fish, and assessment centre region all predicted odds of vitamin D deficiency (<25 nmol/L). High levels of deficiency are concerning due to the known association with poor musculoskeletal health, and potentially increased risk of other chronic health diseases, with associated personal and societal costs. Tailored public health messages for AC and AF may need to focus on year-round vitamin D supplementation and identifying and increasing intakes of vitamin D rich foods. These data also support the urgent requirement for a revised vitamin D RNI for ethnic groups. 

## Figures and Tables

**Table 1:** Categorical demographic and behavioural variables for the African-Caribbean (*n* = 4046) and African (*n* = 2993) UK Biobank participants who had a valid 25(OH)D measurement, including those under the detection limit ^⸸^.

		African-Caribbean			African		
		All *n* = 4046	Male *n* = 1496	Female *n* = 2549	All *n* = 2993	Male *n* = 1536	Female *n* = 1457
Age (at baseline)	40–60	3196 (79.0%)	1155 (77.2%)	2042 (80.1%)	2519 (84.2%)	1306 (85.0%)	1213 (83.3%)
	>60	849 (21.0%)	341 (22.8%)	508 (19.9%)	474 (15.8%)	230 (15.0%)	244 (16.7%)
	*p*	<0.001 ethnic		0.62 gender			0.23 gender
BMI	≤18.5 (underweight)	6 (0.2%)	1 (0.1%)	5 (0.2%)	2 (0.1%)	1 (0.1%)	1 (0.1%)
	18.5–24.9 (healthy)	804 (20.4)	286 (19.6%)	518 (20.9%)	454 (15.7%)	291 (19.6%)	163 (11.5%)
	25–29.9 (overweight)	1605 (40.7%)	712 (48.8%)	893 (36.0%)	1214 (41.9%)	760 (51.3%)	454 (32.1%)
	>30 (obese)	1527 (38.7%)	460 (31.5%)	1067 (43.0%)	1227 (42.4%)	430 (29.0%)	797 (56.3%)
	missing	104 (2.6%)	37 (2.5%)	66 (2.6%)	96 (3.2%)	54 (3.5%)	42 (2.9%)
	*p*	<0.001 ethnic		<0.001 gender			<0.001 gender
Self-reported overall health rating	Excellent/good	2319 (58.0%)	890 (60.2%)	1429 (56.8%)	2035 (70.1%)	1111 (74.3%)	924 (65.6%)
	Fair/poor	1675 (42.0%)	588 (39.8%)	1087 (43.2%)	869 (29.9%)	384 (25.7%)	485 (34.4%)
	Missing	52 (1.3%)	18 (1.2%)	33 (2.3%)	89 (3.0%)	41 (2.7%)	48 (3.3%)
	*p*	<0.001 ethnic		0.03 gender			<0.001 gender
Season of blood draw	Spring	1222 (30.2%)	458 (30.6%)	764 (30.0%)	890 (29.7%)	465 (30.3%)	425 (29.2%)
	Summer	1022 (25.3%)	373 (24.9%)	649 (25.5%)	889 (29.7%)	458 (29.8%)	431 (29.6%)
	Autumn	948 (23.4%)	344 (23.0%)	604 (23.7%)	671 (22.4%)	334 (21.7%)	337 (23.1%)
	Winter	854 (21.1%)	322 (21.5%)	532 (20.9%)	543 (18.1%)	279 (18.2%)	264 (18.1%)
	*p*	<0.001 ethnic			0.90 gender		0.81 gender
Vegetarian	Yes	36 (0.9%)	8 (0.5%)	28 (1.1%)	9 (0.3%)	3 (0.2%)	6 (0.4%)
	No	4010 (99.1%)	1489 (99.5%)	2521 (98.9%)	2984 (99.7%)	1533 (99.8%)	1451 (99.6%)
	Missing	-	-	-	-	-	-
	*p*	<0.001 ethnic		0.07 gender			0.28 gender
Vitamin D containing supplement *	Yes	1508 (37.9%)	432 (29.5%)	1076 (42.8%)	926 (31.6%)	394 (26.2%)	532 (37.2%)
	No	2470 (62.1%)	1033 (70.5%)	1437 (57.2%)	2007 (68.4%)	1110 (73.8%)	897 (62.8%)
	Missing	68 (1.7%)			60 (2.0%)		
	*p*	<0.001 ethnic		<0.001 gender			<0.001 gender
Oily fish consumption	Never	208 (5.2%)	85 (5.8%)	123 (4.9%)	165 (5.8%)	86 (5.9%)	79 (5.6%)
	≤ once per week	1138 (28.6%)	424 (29.0%)	714 (28.3%)	873 (30.5%)	482 (33.1%)	391 (27.8%)
	Once a week	1484 (37.2%)	532 (36.4%)	952 (37.7%)	916 (32.0%)	465 (32.0%)	451 (32.1%)
	2–4 times per week	1038 (26.1%)	371 (25.4%)	667 (26.5%)	716 (25.0%)	336 (23.1%)	380 (27.0%)
	≥ 5 times a week	115 (2.9%)	31 (2.1%)	67 (2.7%)	191 (6.6%)	50 (3.4%)	63 (4.5%)
	Missing	63 (1.6%)			132 (4.4%)		
	*p*	<0.001 ethnic		0.56 gender			0.01 gender
Skin Colour	Brown or black	3762 (93.0%)	1406 (94.0%)	2355 (92.4%)	2640 (88.2%)	1366 (88.9%)	1274 (87.5%)
	Other	284 (7.0%)	90 (6.0%)	194 (7.6%)	352 (11.8%)	170 (11.1%)	1366 (88.9%)
	*p*	<0.001 ethnic		0.06 gender			0.22 gender
Ease of skin tanning	Get very or moderately tanned	2437 (60.2%)	833 (55.6%)	1604 (62.9%)	726 (24.2%)	294 (19.1%)	432 (29.7%)
	Get mildly or occasionally tanned	651 (16.1%)	264 (17.6%)	387 (15.2%)	465 (15.5%)	221 (14.4%)	244 (16.8%)
	Never tan, only burn	266 (6.6%)	123 (8.2%)	143 (5.6%)	561 (18.7%)	326 (21.2%)	235 (16.1%)
	Do not know/prefer not to answer	692 (17.1%)	277 (18.5%)	415 (16.3%)	1240 (41.4%)	695 (45.2%)	545 (37.5%)
	*p*	<0.001 ethnic		<0.001 gender			<0.001 gender
Time spent outdoors in summer (hours)	<1 h	209 (5.9%)	63 (4.7%)	146 (6.6%)	211 (8.2%)	99 (7.5%)	112 (9.0%)
	1–2 h	840 (23.8%)	262 (19.6%)	578 (26.3%)	694 (26.9%)	364 (27.4%)	330 (26.4%)
	3–4 h	1107 (31.3%)	362 (27.0%)	745 (33.9%)	732 (28.4%)	352 (26.5%)	380 (30.4%)
	≥5 h	1379 (39.0%)	652 (48.7%)	727 (33.1%)	942 (36.5%)	513 (38.6%)	429 (34.3%)
	missing	511 (12.6%)			414 (13.8%)		
	*p*	<0.001 ethnic		<0.001 gender			0.03 gender
Use of sun protection	Never/rarely	1656 (40.9%)	769 (51.4%)	887 (34.8%)	1980 (66.2%)	1079 (70.3%)	901 (61.9%)
	Sometimes	1433 (35.4%)	450 (30.1%)	983 (38.6%)	659 (22.0%)	302 (19.7%)	357 (24.5%)
	Most of the time/always	825 (20.4%)	218 (14.6%)	607 (23.8%)	189 (6.3%)	77 (5.0%)	112 (7.7%)
	Do not go out in the sunshine	64 (1.6%)	25 (1.7%)	39 (1.5%)	72 (2.4%)	26 (1.7%)	46 (3.2%)
	Missing	68 (1.7%)	35 (2.3%)	33 (1.3%)	91 (3.0%)	51 (3.3%)	40 (2.7%)
	*p*	<0.001 ethnic		<0.001 gender			<0.001 gender
Income	<£18,000	1034 (33.1%)	353 (30.9%)	681 (34.4%)	847 (37.3%)	435 (35.7%)	412 (39.2%)
	£18,000 to £30,900	908 (29.0%)	306 (26.8%)	602 (30.4%)	653 (28.8%)	340 (27.9%)	313 (29.8%)
	£31,000 to £51,900	741 (23.7%)	272 (23.8%)	469 (23.7%)	490 (21.6%)	285 (23.4%)	205 (19.5%)
	≥£52 000	442 (14.2%)	213 (18.6%)	229 (11.6%)	281 (12.4%)	159 (13.0%)	122 (11.6%)
	Missing	921 (22.8%)			772 (24.1%)		
	*p*	0.01 ethnic		<0.001 gender			0.06 gender
Region	London/South England	2168 (53.6%)	756 (50.5%)	1412 (55.4%)	2022 (67.6%)	992 (64.6%)	1030 (70.7%)
	Midlands/Wales	1159 (28.6%)	456 (30.5%)	703 (27.6%)	289 (9.7%)	161 (10.5%)	128 (8.8%)
	Scotland/North England	719 (17.8%)	285 (19.0%)	434 (17.0%)	682 (22.8%)	383 (24.9%)	299 (20.5%)
	*p*	<0.001 ethnic		0.01 gender			<0.001 gender
Born UK/ROI	No	2351 (58.1%)	878 (58.7%)	1473 (57.8%)	2657 (88.8%)	1392 (90.6%)	1265 (86.8%)
	Yes	1674 (41.4%)	612 (40.9%)	1062 (41.7%)	271 (9%)	115 (7.5%)	156 (10.7%)
	Missing	21 (0.5%)	7 (0.5%)	14 (0.5%)	65 (2.2%)	29 (1.9%)	36 (2.5%)
	*p*	<0.001 ethnic		0.79 gender			0.02 gender
Oral contraceptive use (females)	Yes	-	-	82 (3.2%)	-	-	34 (2.3%)
	No	-	-	980 (38.4%)	-	-	581 (39.9%)
	Missing	-	-	1487 (58.3%)	-	-	842 (57.8%)
	*p*	<0.001 ethnic					
Menopause (females)	Yes	-	-	1043 (40.9%)	-	-	679 (46.6%)
	No	-	-	989 (38.8%)	-	-	551 (37.8%)
	Not sure (had a hysterectomy)	-	-	344 (13.5%)	-	-	104 (7.1%)
	Not sure (other reason)/Prefer not to say			152 (6.0%)			152 (6.0%)
	Missing	-	-	1497 (37.0%)	-	-	1537 (51.4%)
	*p*	<0.001 ethnic					
Current tobacco smoker	Yes/occasionally	676 (16.7%)	357 (23.8%)	319 (12.5%)	206 (6.9%)	159 (10.4%)	47 (3.2%)
	No	3358 (83.0%)	1136 (75.9%)	2222 (87.2%)	2781 (92.9%)	1372 (89.3%)	1409 (96.7%)
	Missing	12 (0.3%)	4 (0.3%)	8 (0.3%)	6 (0.2%)	5 (0.3%)	1 (0%)
	*p*	<0.001 ethnic		<0.001 gender			<0.001 gender
Inflammatory condition **	Yes	189 (4.7%)	72 (4.8%)	117 (4.6%)	87 (2.9%)	49 (3.2%)	38 (2.6%)
	No	3857 (95.3%)	1424 (95.2%)	2432 (95.4%)	2906 (97.1%)	1487 (96.8%)	1419 (97.4%)
	*p*	<0.001 ethnic		0.74 gender			0.34 gender

BMI = body mass index, UK = United Kingdom, ROI = Republic of Ire-land. All *p*-values are chi-square. All percentages are valid percentages (i.e., excluded missing data).⸸ recorded as 7.1 nmol/L; * Vitamin D containing supplement means either single vitamin D supplement or multivitamin which contains vitamin D [14].** Inflammatory conditions refer to the most common conditions: Rheumatoid arthritis, inflammatory bowel disease (Crohns and Ulcerative Colitis), Chronic obstructive pulmonary disease and Systemic Lupus Erythematosis (SLE).

**Table 2:** Median 25(OH)D concentration and vitamin D cut-offs of the African-Caribbean (*n* = 4046), African (*n* = 2993), White (*n* = 3986) and South Asian (*n* = 7256) participants from the UK Biobank.

Group	*n*	Median 25(OH)D (nmol/L)	IQR 25(OH)D (nmol/L)	*p*-Value for Medians *	25(OH)D Deficient (<25 nmol/L)	25(OH)D Insufficient (>25–50 nmol/L)	25(OH)D Sufficient (>50 nmol/L)
All African-Caribbean	4046	30.0	20.9	<0.001 ethnicities ^†^ *p* = 0.41 AC vs. AF ^†^ *p* < 0.001 for all other ethnicity vs. ethnicity post hoc tests	1499 (37.0%)	1906 (41.1%)	642 (15.9%)
African-Caribbean Male	1497	29.5	19.3	0.01 gender	604 (40.3%)	676 (45.2%)	217 (14.5%)
African-Caribbean Female	2549	30.4	21.5		895 (35.1%)	1230 (48.2%)	424 (16.7%)
All African	2993	30.2	20.0		1042 (34.8%)	1539 (51.4%)	412 (13.8%)
African Male	1536	28.5	19.1	<0.001 gender	589 (38.3%)	773 (50.3%)	174 (11.3%)
African Female	1457	32.0	20.9		453 (31.1%)	766 (52.6%)	238 (16.3%)
All White (random sample)	3986	49.2	29.5		459 (11.5%)	1591 (39.9%)	642 (15.9%)
White Male	1910	49.2	29.7	0.47 gender	201 (10.5%)	780 (40.8%)	929 (48.6%)
White Female	2076	49.3	29.1		258 (12.4%)	811 (39.0%)	1007 (48.5%)
All South Asian	7256	20.7	18.5		4454 (61.4%)	2280 (31.4%)	523 (7.2%)
South Asian Male	3922	20.1	16.7	<0.001 gender	2517 (64.2%)	1202 (30.6%)	203 (5.2%)
South Asian Female	3334	21.8	21.1		1936 (58.1%)	1078 (32.3%)	320 (9.6%)

All percentages are valid percent.* *p*-Values were analysed using Mann–Whitney for African-Caribbean male vs. female and African male vs. female. Kruskal Wallis H test for African-Caribbean vs. African vs. White vs. South Asian. ^†^ *p*-Values were analysed using Mann–Whitney for African-Caribbean vs. African, African-Caribbean vs. White, African-Caribbean vs. South Asian, African vs. South Asian, African vs. White, White vs. South Asian. Post hoc comparisons were statistically significant (*p* < 0.001), except African-Caribbean vs. African (*p* = 0.41). Bonferroni adjustment with a revised statistically significant *p*-value of *p* < 0.008.

**Table 3:** Serum 25(OH)D by season and ethnicity for the African-Caribbean (*n* = 4046) and African (*n* = 2993) participants from the UK Biobank.

Group	Spring			Summer			Autumn			Winter			*p*-Value for Medians *
	*n*	Median (nmol/L)	IQR (nmol/L)	*n*	Median (nmol/L)	IQR (nmol/L)	*n*	Median (nmol/L)	IQR (nmol/L)	*n*	Median (nmol/L)	IQR (nmol/L)	
African-Caribbean (*n* = 4046)	1222 (30.2%)	27.1	19.5	1022 (25.3%)	34.8	22.3	948 (23.4%)	31.7	20.7	854 (21.1%)	26.1	18.5	Ethnicity × season *p* < 0.001
African-Caribbean male (*n* = 1496)	458 (30.6%)	25.3	17.7	373 (24.9%)	35.2	23.9	344 (23.0%)	30.9	19.2	322 (21.5%)	24.2	17.7	Gender × season *p* = 0.11 *Season main effects: p* < 0.001 *Gender main effects: p* = 0.02
African-Caribbean female (*n* = 2549)	764 (30.0%)	27.7	21.2	649 (25.5%)	34.5	21.7	604 (23.7%)	31.8	22.1	532 (20.9%)	26.7	19.0	
African (*n* = 2993)	890 (29.7%)	28.3	19.7	889 (29.7%)	33.8	20.3	671 (22.4%)	31.5	19.5	543 (18.1%)	26.8	18.9	
African male (*n* = 1536)	465 (30.3%)	27.4	18.8	458 (29.8%)	31.6	19.6	334 (21.7%)	31.2	18.6	279 (18.2%)	24.7	16.0	Gender × season *p* = 0.51 *Season main effects: p* < 0.001 *Gender main effects: p* < 0.001
African female (1457)	425 (29.2%)	30.3	21.0	431 (29.6%)	36.3	21.8	337 (23.1%)	31.8	21.25	264 (18.1%)	29.5	20.5	

* *p*-Values from two-way ANOVA. Note: Each participant has one measurement in one season, therefore data does not show repeated measures. There was a statistical significance between ethnicities and also between seasons. Both groups reported the highest median 25(OH)D concentrations in summer and the lowest in winter.

**Table 4:** 25(OH)D (nmol/L) by geographical location of the African-Caribbean (*n* = 4046) and African (*n* = 2993) UK Biobank participants.

	London/South England Latitude: London: *Bart’s Croydon and Hounslow* 51.4° N to 51.5° N South England: *Bristol, Oxford and Reading* 51.5° N to 51.8° N			Midlands/Wales Latitude: Midlands: *Birmingham, Nottingham, Stoke on Trent* 52.5° N to 53° N Wales: *Cardiff, Swansea, Wrexham* 51.5° N to 53.0° N			Scotland/North England Latitude: Scotland: *Glasgow and Edinburgh* 55.9° N to 56.0° N North England: *Manchester, Stockport, Bury, Leeds, Liverpool, Middlesbrough, Newcastle, Sheffield, Cheadle* 53.4° N to 55° N			*p*-Values for Medians *
	*n*	Median	IQR	*n*	Median	IQR	*n*	Median	IQR	
All African-Caribbean (*n* = 4046)	2168	31.0	22.0	1159	27.5	19.3	719	30.1	19.0	Ethnicity × geographical location *p* < 0.001
African-Caribbean male (*n* = 1496)	756	30.9	20.8	456	26.4	18.3	285	29.6	18.1	*p* = 0.725 gender × geographical location *Geographical main effects: p < 0.001* *Gender main effects: p = 0.049*
African-Caribbean female (*n* = 2549)	1412	31.3	21.9	703	28.2	20.4	434	30.6	20.5	
All African (*n* = 2993)	2022	32.3	21.4	289	25.9	16.4	682	27.4	16.55	
African male (*n* = 1536)	992	30.9	20.2	161	24.7	16.6	383	26.3	14.6	*p* = 0.624 gender × geographical location *Geographical main effects: p < 0.001* *Gender main effects: p = 0.002*
African female (*n* = 1457)	1030	33.8	21.5	128	26.9	18.1	299	28.3	17.5	

* *p*-Values two-way ANOVA. Latitudes according to UK Biobank assessment centres.

**Table 5:** Prediction of vitamin D deficiency (<25 nmol/L) in the UK Biobank African-Caribbean participants.

Model		*n*	B	SE	*p*	Odds Ratio	95% CI for Odds Ratio	
							Lower	Upper
Model 1 *N* = 3892 *p* < 0.001 Nagelkerke R2 = 0.041 HL test, *p* = 0.496	Gender							
	*Female (reference)*	2442						
	*Male*	1450	0.27	0.07	<0.001	1.31	1.14	1.50
	Age							
	*40–60 (reference)*	3090						
	*>60*	802	−0.82	0.09	<0.001	0.44	0.37	0.53
	BMI							
	*≤25.4 (normal/underweight) (reference)*	926			0.01			
	*25–29.4 (overweight)*	1307	−0.12	0.09	0.16	0.88	0.74	1.05
	*>30 (obese)*	1659	0.12	0.09	0.16	1.13	0.95	1.33
	Skin colour							
	*Other (reference)*	225						
	*Brown or black*	3667	0.47	0.16	<0.001	1.60	1.18	2.18
Model 2 *n* = 3764 *p* <0.001 Nagelkerke R2 = 0.12 HL test, *p* = 0.828	Gender							
	*Female (reference)*	2381						
	*Male*	1383	0.15	0.07	0.04	1.16	1.00	1.34
	Age							
	*40–60 (reference)*	3001						
	*>60*	763	−0.89	0.10	<0.001	0.41	0.34	0.50
	BMI							
	*≤25.4 (normal/underweight) (reference)*	899			0.01			
	*25–29.4 (overweight)*	1262	−0.14	0.10	0.15	0.87	0.72	1.05
	*>30 (obese)*	1603	0.12	0.09	0.20	1.12	0.94	1.34
	Skin colour							
	*Other (reference)*	217						
	*Brown or black*	3547	0.53	0.17	<0.001	1.70	1.23	2.36
	Oily fish intake							
	*Yes (reference)*	3597						
	*No*	167	0.52	0.17	<0.001	1.69	1.22	2.34
	Vitamin D supplementation							
	*User (reference)*	1441						
	*Non- user*	2323	1.09	0.08	<0.001	2.98	2.56	3.47
Model 3 *n* = 2955 *p* < 0.001 Nagelkerke R2 = 0.16 HL test, *p* = 0.804	Gender							
	*Female (reference)*	1879						
	*Male*	1077	0.11	0.09	0.22	1.11	0.94	1.31
	Age							
	*40–60 (reference)*	2498						
	*>60*	458	−0.75	0.13	<0.001	0.47	0.37	0.61
	BMI							
	*≤25.4 (normal/underweight) (reference)*	683			0.02			
	*25–29.4 (overweight)*	1020	−0.08	0.11	0.45	0.92	0.74	1.14
	*>30 (obese)*	1252	0.17	0.11	0.11	1.18	0.96	1.45
	Skin Colour							
	*Other (reference)*	146						
	*Brown or black*	2809	0.57	0.20	0.01	1.77	1.19	2.63
	Oily fish intake							
	*Yes (reference)*	2828						
	*No*	127	0.55	0.19	<0.001	1.74	1.19	2.54
	Vitamin D supplementation							
	*User (reference)*	1147						
	*Non-user*	1808	1.09	0.09	<0.001	2.97	2.50	3.53
	Region							
	*London/South England (reference)*	1603			0.10			
	*Midlands/Wales*	828	0.19	0.09	0.04	1.22	1.01	1.46
	*Scotland/North England*	524	0.14	0.11	0.22	1.15	0.92	1.43
	Season of blood draw							
	*Spring (reference)*	897			<0.001			
	*Summer*	744	-1.03	0.12	<0.001	0.36	0.28	0.45
	*Autumn*	673	−0.46	0.11	<0.001	0.63	0.51	0.79
	*Winter*	641	0.20	0.11	0.07	1.22	0.98	1.51
	Income							
	*£18,000*	956			0.97			
	*£18,000–30,999*	857	−0.04	0.11	0.71	0.96	0.78	1.18
	*£31,000–51,999*	714	0.01	0.11	0.94	1.01	0.81	1.26
	*≥£52,000*	428	−0.02	0.13	0.91	0.99	0.76	1.27

HL = Hosmer–Lemeshow test, B = unstandardised coefficient, SE = standard error, CI = confidence intervals. Odds ratio is the odds of having serum 25(OH)D <25 nmol/L. BMI units kg/m^2^. Sun protection refers to use of sunscreen or a hat. Vitamin D supplementation refers to either single vitamin D supplements or multivitamins which contains vitamin D [14].

**Table 6:** Median and IQR values from the 24 h recall, for the vitamin D intakes (*n* = 1343 African-Caribbean and *n* = 850 African participants with measurements) and calcium intakes (*n* = 1491 African-Caribbean and *n* = 960 African participants with measurements) for those African-Caribbean and African participants who had a valid 25(OH)D measurement in the UK Biobank.

	Vitamin D Intake							Calcium Intake						
Group	*n*	Missing	Median (µg/day)	IQR (µg/day)	Suboptimal Intake (<10 µg/day)	Optimal Intake (>10 µg/day)	*p*-Value for Medians *	*n*	Missing	Median (mg/day)	IQR (mg/day)	Suboptimal Intake (<700 mg/day)	Optimal Intake (>700 mg/day)	*p*-Value for Medians *
All African-Caribbean	1344 (33.2%)	2703 (66.8%)	1.6	2.6	1280 (95.2%)	64 (4.8%)	<0.001 ethnicities	1343 (33.2%)	2703 (66.8%)	726.8	536.2	641 (47.7%)	702 (52.3%)	<0.001 ethnicities
African-Caribbean Male	454	-	1.8	2.8	429 (94.5%)	25 (5.5%)	0.13 gender	454	-	741.6	516.4	209 (46.0%)	245 (54.0%)	0.05 gender
African-Caribbean Female	890	-	1.5	2.5	851 (95.6%)	39 (4.4%)		889	-	721.9	543.6	432 (48.6%)	457 (51.4%)	
All African	850 (28.4%)	2143 (71.6%)	2.1	4.2	773 (90.9%)	77 (9.1%)		850 (28.4%)	2143 (71.6%)	730.7	579.1	404 (47.5%)	446 (52.5%)	
African Male	461	-	2.2	4.6	412 (89.4%)	49 (10.6%)	0.02 gender	461	-	741.9	613.0	220 (47.7%)	241 (52.3%)	0.87 gender
African Female	389	-	1.9	3.8	361 (92.8%)	28 (7.2%)		389	-	729.6	552.5	184 (47.3%)	205 (52.7%)	
All White (random sample)	1578 (39.6%)	2409 (60.4%)	1.9	2.3	1514 (95.9%)	64 (4.1%)		1577 (39.6%)	2409 (60.4%)	943.9	429.8	337 (21.4%)	1240 (78.6%)	
White Male	746	-	2.0	2.3	714 (95.7%)	32 (4.3%)	0.01 gender	746	-	992.9	473.7	135 (18.1%)	611 (81.9%)	<0.001 gender
White Female	832	-	1.77	2.1	800 (96.2%)	32 (3.8%)		831	-	902.4	388.0	202 (24.3%)	629 (75.7%)	
All South Asian	2033 (28.0%)	5224 (72.0%)	1.1	1.8	1973 (97.0%)	60 (3.0%)		2033 (28.0%)	5223 (72.0%)	826.3	543.7	747 (36.7%)	1286 (63.3%)	
South Asian Male	1099	-	1.2	1.9	1061 (96.5%)	38 (3.5%)	<0.001 gender	1099	-	863.6	569.2	364 (33.1%)	735 (66.9%)	<0.001 gender
South Asian Female	934	-	1.1	1.7	912 (97.6%)	22 (2.4%)		934	-	782.9	514.0	383 (41.0%)	551 (59.0%)	

* *p*-Values were analyzed using Mann–Whitney for African-Caribbean male vs. female and African male vs. female, Kruskal–Wallis H test for African-Caribbean vs. African vs. White vs. South Asian.

## Data Availability

We do not have permission to share UK Biobank data and interested persons should contact the UK Biobank directly if they wish to access the data.
